# Healthcare value of implementing hepatitis C screening in the adult general population in Spain

**DOI:** 10.1371/journal.pone.0208036

**Published:** 2018-11-28

**Authors:** María Buti, Raquel Domínguez-Hernández, Miguel Ángel Casado, Eliazar Sabater, Rafael Esteban

**Affiliations:** 1 Hospital General Universitario Vall d'Hebron, CIBERehd, Barcelona, Spain; 2 Pharmacoeconomics & Outcomes Research Iberia, Madrid, Spain; National Taiwan University Hospital, TAIWAN

## Abstract

**Background:**

Elimination of hepatitis C virus (HCV) infection requires high diagnostic rates and universal access to treatment. Around 40% of infected individuals are unaware of their infection, which indicates that effective screening strategies are needed. We analyzed the efficiency (incremental cost-utility ratio, ICUR) of 3 HCV screening strategies: a) general population of adults, b) high-risk groups, and c) population with the highest anti-HCV prevalence plus high-risk groups.

**Methods:**

An analytical decision model, projecting progression of the disease over a lifetime, was used to establish the candidate population for HCV screening. HCV data were obtained from the literature: anti-HCV prevalence (0.56%-1.54%), viremic patients (31.5%), and percentage of undiagnosed persons among those with viremia (35%). It was assumed that most patients would be treated and have HCV therapy response (98% SVR); transition probabilities, utilities, and disease management annual costs were obtained from the literature. Efficiency over the life of patients under the National Health System perspective was measured as quality-adjusted life years (QALY) and total cost (screening, diagnosis, pharmacological and disease management). A discount rate of 3% was applied to costs and outcomes.

**Results:**

Screening of the adult population would identify a larger number of additional chronic hepatitis C cases (N = 52,694) than screening the highest anti-HCV prevalence population plus high-risk groups (N = 42,027) or screening high-risk groups (N = 26,128). ICUR for the general population vs. high-risk groups was €8914/QALY gained per patient (€18,157 incremental cost and 2.037 QALY). ICUR for the general population vs. population with highest anti-HCV prevalence plus high-risk groups was €7,448/QALY gained per patient (€7,733 incremental cost and 1.038 QALY). These ICUR values are below the accepted efficiency threshold (€22,000-€30,000).

**Conclusion:**

HCV screening and treatment of the general adult population is cost-effective compared to screening of high-risk groups or the population with the highest anti-HCV prevalence plus high-risk groups.

## Introduction

The World Health Organization has recommended elimination of hepatitis C virus (HCV) as a public health threat in 2030 [1]. Hepatitis C is the most prevalent viral hepatitis in Western countries. Recent epidemiological studies performed in Spain show that the prevalence of active HCV (ie, HCV RNA positive status) ranges from 0.35% to 0.41% of adults in the general population [2–4]. Most individuals with chronic hepatitis C (CHC) are asymptomatic and manifest clinical symptoms only in advanced phases of the disease, which makes it difficult to diagnose this condition in early stages [5]. CHC patients can develop liver cirrhosis, decompensated cirrhosis, or hepatocellular carcinoma [5]. This last complication is a major cause of liver cancer in Spain [6]. Oral direct-acting antiviral (DAA) agents have changed the therapeutic scenario of CHC, allowing HCV cure in almost all patients regardless of HCV genotype or degree of liver fibrosis. In addition, the safety profile is excellent even in patients with decompensated liver disease. [5].

To enable DAA treatment access to the largest number of patients with hepatitis C in Spain, the Strategic Plan for Tackling Hepatitis C (PEAHC, *Plan Estratégico para el Abordaje de la Hepatitis* C) [7] was developed within the Spanish National Health System. This plan initially contemplated priority for patients in advanced stages of the disease, but now the health system reimburses treatment for all patients with CHC, regardless of their degree of fibrosis or previous treatment experience [8].

The PEAHC has allowed treatment for a large number of patients, but many affected individuals do not know that they have HCV infection. Currently, HCV screening of the general population is not recommended in Spain or in most other countries, and is limited to persons in high-risk groups, such as people who inject drugs, men who have sex with men, prisoners, and patients receiving hemodialysis [7, 9–11]. However, this approach does not suffice to eliminate hepatitis C in a specific geographical area. There is a need to identify and treat the HCV-unknown population to reduce the number of infective individuals and avoid transmission of the disease and re-infection [12].

Chronic hepatitis C also has a large economic impact [13]; hence, cost evaluations are needed to assess the clinical and economic burden, and define the most effective screening programs for this disease.

The objective of this study was to estimate the cost-utility of screening and treatment of the general adult population and compare this approach with two other screening strategies: screening of individuals in high-risk groups (the currently recommended strategy) and screening of adults with the highest prevalence of HCV antibodies (anti-HCV) plus high-risk groups.

## Methods

A decision-analytic model and a Markov model were used to evaluate the cost-utility of 3 different HCV screening strategies involving a single anti-HCV determination: 1) screening of adults in the general population (individuals 20–79 years of age or born in 1938–1997); 2) screening of high-risk groups aged 20–79 years (born, 1938–1997) according to current recommendations (People Who Inject Drug (PWID), prisoners, HIV/HCV coinfected); and 3) screening of the population with the highest prevalence of anti-HCV, aged 50–79 years (born, 1938–1967) plus high-risk groups (20–49 years; born, 1968–1997). In the analysis, the general population was compared with the high-risk groups (scenario 1) and with the population with the highest anti-HCV prevalence plus high-risk groups (scenario 2).

The main outcomes of the analysis were to evaluate the clinical benefits of screening, including the number of cases of liver-related complications avoided and the patients’ quality-adjusted life (QALY), and the economic benefits estimated by the incremental cost-utility ratio, expressed as the incremental cost per QALY gained (For more details, refer to S1 Text). The evaluation was performed from the perspective of the Spanish National Health System. The total cost calculated for each strategy was the sum of the cost of screening and diagnosing patients with chronic hepatitis C plus the lifetime healthcare cost related to chronic hepatitis C patients. Costs and health outcomes were considered over a lifetime horizon and were discounted at 3% annually [14].

### Decision tree

**Fig 1:**
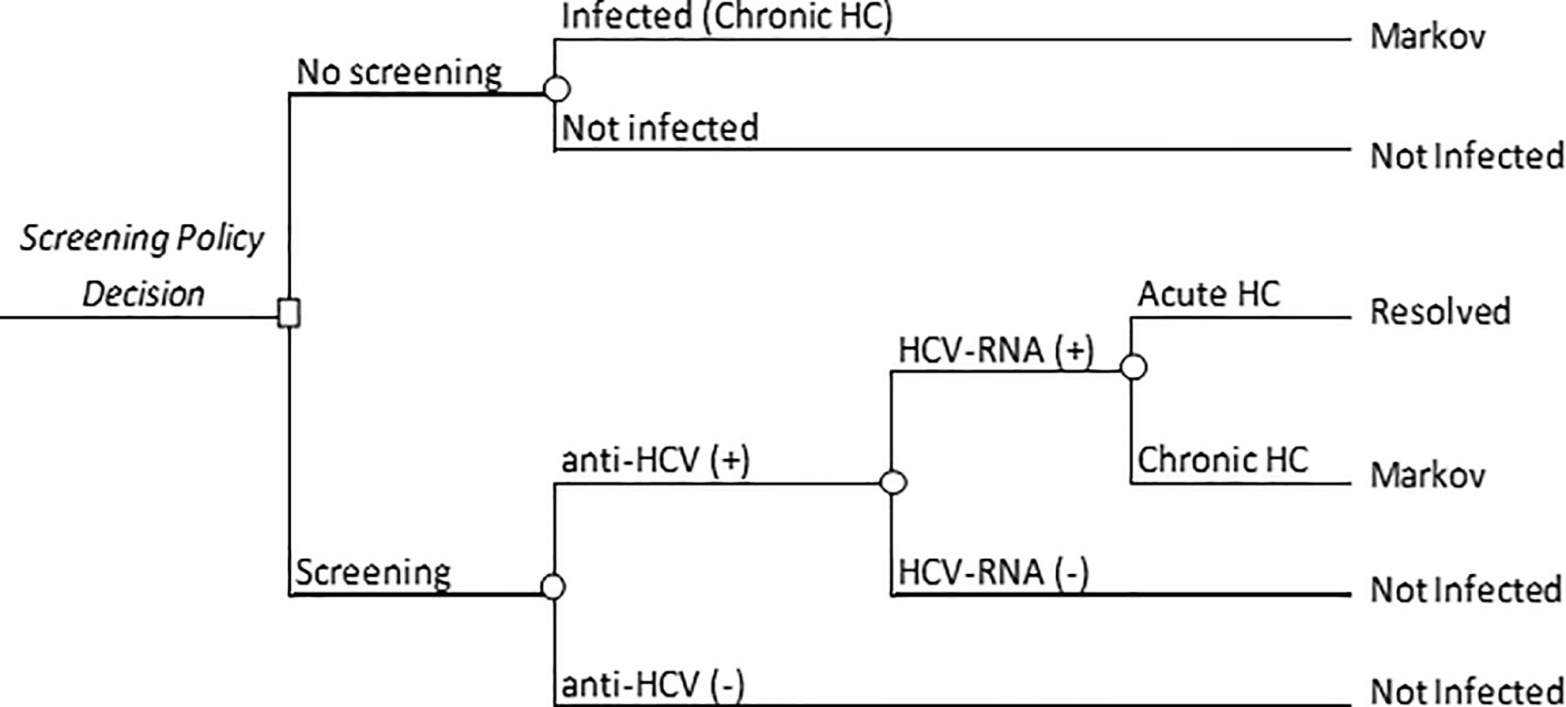
Decision tree. HC, hepatitis C; HCV, hepatitis C virus; RNA +, ribonucleic acid positive; RNA -, ribonucleic acid negative. Decision tree showing the decision of whether or not to screen. Target populations were tested only once at the beginning of the analysis. The population eligible for screening was estimated after excluding the population already diagnosed with HCV infection. All screened patients were assumed to undergo antibody testing by ELISA (enzyme-linked immunosorbent assay), following by polymerase chain reaction (PCR) in those testing antibody-positive to confirm the diagnosis of the disease. In patients testing positive on ELISA but negative on PCR, it was assumed that the infection had resolved or spontaneously cleared. Only chronic hepatitis C patients were entered in the Markov model and progressed in the disease until death.

The decision-analytic model used to evaluate the 3 approaches reflects the choice of each HCV screening strategy and estimates the chronic hepatitis C population diagnosed and treated in each of them (Fig 1).

**Fig 2:**
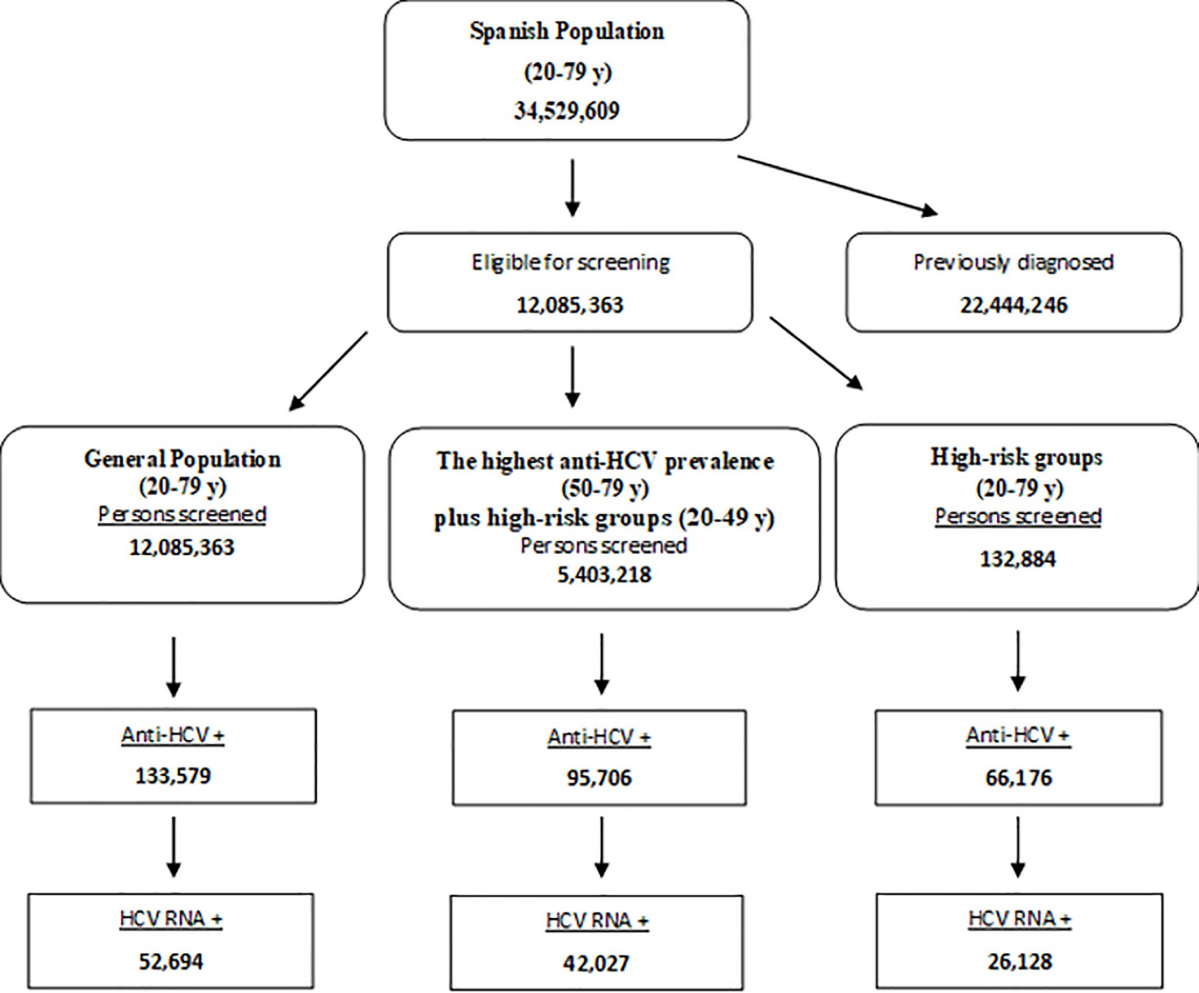
Screening flow chart. Screening flow chart showing the derivation of the number of individuals screened, HCV-diagnosed, eligible for treatment, and achieving SVR in the general population, the highest anti-HCV prevalence population plus high-risk groups, and high-risk-groups.

The Spanish adult population value was obtained from the registry data of the Spanish population [15]. The number of patients with previously diagnosed chronic hepatitis C is shown in Fig 2. Undiagnosed individuals with HCV infection (35% of the Spanish population) [3] were eligible for screening, and 100% of them were assumed to be tested. The general adult population was broken down into 3 age groups because of the differing prevalence of HCV antibodies and HCV RNA according to this factor: 20 to 34 years, 35 to 49 years, and 50 to 79 years (Table 1) [3]. The population with the highest prevalence of anti-HCV was the 50 to 79 year-old group. The population of high-risk individuals was estimated from data reported in several Spanish studies (Fig 2) [16–25].

**Table 1:** Values of the parameters in the model.

Variable	Baseline	Reference
Patient characteristics at time of screening		
Total population	34,529,609	INE
*1983–1997 (20–34 years)*	*7*,*868*,*439*	INE
*1968–1982 (35–49 years)*	*11*,*463*,*451*	INE
*1938–1967 (50–79 years)*	*15*,*197*,*719*	INE
Prevalence of anti-HCV positive, %		
1983–1997 (20–34 years)	0.56 (0.03–1.60)	[3]
1968–1982 (35–49 years)	1.11 (0.82–1.49)	[3]
1938–1967 (50–79 years)	1.54 (1.18–1.193)	[3]
HCV RNA (viral load) detected, %		
1983–1997 (20–34 years)	100.0	[3]
1968–1982 (35–49 years)	34.8	[3]
1938–1967 (50–79 years)	30.6	[3]
**Natural history disease parameters**		
**Disease progression (Transition probability)**		
F0 to F1 (age 20–29 y)	0.314	[28]
F0 to F1 (age 30–49 y)	0.131	[28]
F0 to F1 (age 50+ y)	0.077	[28]
F1 to F2 (age 20–29 y)	0.322	[28]
F1 to F2 (age 30–49 y)	0.080	[28]
F1 to F2 (age 50+ y)	0.074	[28]
F2 to F3 (age 20–29 y)	0.220	[28]
F2 to F3 (age 30–49 y)	0.133	[28]
F2 to F3 (age 50+ y)	0.089	[28]
F3 to F4 (age 20–29 y)	0.151	[28]
F3 to F4 (age 30–49 y)	0.134	[28]
F3 to F4 (age 50+ y)	0.088	[28]
F3 to HCC	0.011	[33]
SVR after F3 to HCC	0.003	[33]
F4 to DC	0.040	[32]
F4 to HCC	0.015	[27]
SVR after F4 to Regr. HC	0.055	[30]
SVR after F4 to DC	0.003	[33]
SVR after F4 to HCC	0.005	[33]
DC to HCC	0.068	[31]
DC to LT	0.023	[29]
HCC to LT	0.040	[29]
LT to post-LT	1.000	Assumption*
**Liver-related mortality, annual probability**		
DC	0.133	[31]
HCC	0.430	[29]
LT	0.210	[27]
Post-LT	0.057	
**Utilities**		
F0	0.98	[28]
F1	0.98	[28]
F2	0.92	[28]
F3	0.79	[28]
F4	0.76	[28]
SVR after F0	1.00	[28]
SVR after F1	1.00	[28]
SVR after F2	0.93	[28]
SVR after F3	0.86	[28]
SVR after F4	0.83	[28]
Regr. HC	0.86	Assumption^‡^
DCC	0.69	[28]
CHC	0.67	[28]
LT	0.50	[28]
Post-LT	0.77	[28]
**Cost related to screening and diagnosis, € (2017)**		
Blood test ^a^	€41	[39]
Diagnosis (ELISA and PCR test)	€30 and €74	[39]
Fibroscan	€21	[38]
Diagnosis ^b^	€508	
**Annual health state cost, € (2017)**		
F0	€272	[41]
F1	€272	[41]
F2	€314	[41]
F3	€314	[41]
F4	€572	[41]
SVR after F0	€115	[32]
SVR after F1	€115	[32]
SVR after F2	€115	[32]
SVR F0, F1, F2 su*bsequent years*	€0	Assumption^#^
SVR after F3	€115	[32]
SVR F3 su*bsequent years*	€115	Assumption^#^
SVR after F4	€166	[32]
SVR F4 su*bsequent years*	€166	Assumption^#^
Regr. HC	€115	Assumption^#^
DC	€2,332	[41]
CHC	€8,884	[41]
LT	€125,294	[41]
Post-LT		
*1*^*st*^ *year*	€36,622	[41]
*Subsequent years*	€18,311	[41]

BC, birth cohort; CHC, chronic hepatitis C; DC, decompensated cirrhosis; ELISA, enzyme-linked immunosorbent assay; F, Metavir fibrosis score; HCC, hepatocellular carcinoma; HCV, hepatitis C virus; LT, liver transplantation; PCR, polymerase chain reaction; Regr. HC, regression of hepatic cirrhosis; SVR, sustained virologic response 12 weeks after treatment.

^a^ One-time only screening test.

^b^ Other costs related to the diagnosis. ELISA and PCR to confirm chronic hepatitis C were not included.

*Patients with successful liver transplant achieve a clinical improvement over time. Therefore, these patients only remained one cycle in LT stage and then they progress to post-LT stage until their deaths.

^‡^Fibrosis regression is defined as a reduction of at least 1 point in Metavir fibrosis score. For Regr. HC state, the utility value of SVR F3 was assumed.

^#^Patients with a prolonged response over time achieve disease cure. Therefore, it was assumed that no direct medical costs incurred by patients in SVR F0, SVR F1 and SVR F2 state in the second year and subsequences.

HCV screening consisted of anti-HCV antibody testing in a single determination. Individuals testing positive were then further tested for HCV RNA, and those with viremia were eligible for therapy [9–11].

Individuals with HCV RNA and those who were unaware of their HCV infection status were stratified to populate the Markov model. Uninfected individuals were not included in the model.

### Markov model

The natural history of chronic hepatitis C was simulated with a Markov model based on a previously validated model [26]. Patients with chronic hepatitis C infection entered the model distributed across fibrosis stages (52.5% F0-F1, 12.5% F2, 12.5% F3, and 12.5% F4) [4] according to the average age of each age group. They could then progress in annual cycles through the other liver health states (decompensated cirrhosis [DC], hepatocellular carcinoma [HCC], and liver transplantation [LT]) to death. LT patients remained in this state for only 1 cycle before they transited to post-LT state. Post-LT patients remained in this state up to death. Annual health state transition probabilities were estimated using data from the literature [27–33] (Table 1). (See S2 Text, S1 Fig and S1 Table for a detailed description).

In the Markov model, newly diagnosed patients with chronic hepatitis C received treatment (82%) [34] with a pangenotypic DAA regimen. An SVR rate of 98% was assumed for all treated patients regardless of their fibrosis stages (F0-F4), based on data from the literature [35]. Discontinuation of treatment and adverse events (AEs) were not considered in the analysis, as the related literature has reported very low treatment discontinuation rates and no severe medication-related AEs associated with these drugs.

In strategies 2 and 3, individuals who are not screened may have HCV infection, but are unaware of their status. These patients, those who are ineligible for therapy, and those failing treatment progress according to the natural history of the disease.

Spanish mortality tables by age groups were used to estimate age-specific mortality rates for all patients [36]. The overall population mortality rates were adjusted by the liver-related mortality rates reported in Spanish chronic hepatitis C patients [37]. (See S2 Table for more details on mortality rates).

Health utility values adopted in previous economic evaluations were applied for each health state [28] (Table 1).

### Costs

Because of the analysis perspective, only direct costs were considered. All costs were based on the 2017 values.

The cost of HCV screening was that of a single HCV blood test during a medical check-up. Costs associated with the HCV diagnosis were obtained from the Spanish database of medical costs [38, 39] (Table 1).

The DAA regimen cost per patient was calculated based on the overall cost of chronic hepatitis C treatment (€24,167) and the total of chronic hepatitis C patients treated with DAAs during one year in Spain [40]. The monitoring cost (€1328) was obtained from the Spanish literature [41].

The costs of the various liver health states were derived from previously published literature specific to Spain and adjusted to the 2017 consumer price index data [32, 41] (Table 1). Patients who achieved SVR and had initially mild fibrosis (F0-F2) were discharged and were assumed not to incur any new cost for the health system. However, patients with SVR in fibrosis stages F3 or F4 were considered to require monitoring for HCC and had additional costs related to the disease management.

HCV-unaware patients belonging to the high-risk groups were assumed to be diagnosed when they had hepatic decompensation or HCC, and from that point on began to generate costs.

### Sensitivity analyses

Uncertainties in the estimates applied to some parameters with greater impact on the analysis results were assessed in a one-way sensitivity analysis. The variation for the parameters included was taken from the literature, or was set at ±20% when no related information was available. The sensitivity analysis model was adjusted to allow variations in the following parameters: percentage of previously undiagnosed patients with HCV infection (30%-52%), prevalence of individuals with anti-HCV (+) status (95% CI) [3], fibrosis distribution (±20% for F4), percentage of patients treated (90% was assumed), SVR rates (95% CI) [35], progression rate from DC to LT and from HCC to LT, and QALY from liver disease states. The costs of anti-HCV testing (±20%), treatment and disease management (±20%), and discount rates (0%-5%) were also included.

In addition, we evaluated the impact on health and economic outcomes of increasing the percentage of patients initially treated to 95% or 100%, and determined the various ranges of values for treatment costs assuming a decrease of 10%, 20% or 30%.

## Results

### Population

The total number of adults (>20 years) currently living in Spain was estimated at 34,529,609 individuals. Excluding 22,444,246 individuals already diagnosed with HCV, the numbers considered for HCV screening were 12,085,363, for the general population, 5,403,218 for highest anti-HCV prevalence plus high-risk groups, and 132,884 for high-risk groups. Based on these figures, HCV RNA was detected by screening in 52,694 individuals from general population, 42,027 from the highest anti-HCV prevalence plus high-risk groups, and 26,128 from the high-risk groups (Fig 2).

As to patients with HCV viremia considered for therapy, there were 43,209 from the general population (42,691 achieving SVR), 34,462 from the highest anti-HCV prevalence plus high-risk groups (34,049 with SVR), and 21,425 from the high-risk groups (21,168 achieving SVR).

The results of the analysis in the general population indicate that screening of 12,085,363 persons would detect 52,694 patients with viremia; that is, detection of 1 case per every 229 individuals screened.

### Screening cost

The incremental cost of testing to identify one case of HCV by routine screening of the general population was €8418 (€8511 vs €94) in scenario 1, and €4706 (€8511 vs €3805) in scenario 2.

### Cost-utility analysis

In scenario 1, the incremental cost-utility ratio (ICUR) per patient associated with providing one-time screening and treatment in HCV RNA-positive patients eligible for therapy for the general population versus the high-risk groups was €8914/QALY. In scenario 2, the ICUR per patient when comparing the general population versus the population with the highest anti-HCV prevalence plus high-risk groups was €7448/QALY (Table 2). Both scenarios were associated with ICURs below the accepted range of €22,000 to €30,000 per QALY [42, 43], suggesting that addition of one-time screening is cost-effective in both strategies.

**Table 2:** Target population results and cost-utility results per patient (discounted).

	QALY	Costs	Incremental Cost	Incremental QALY	ICUR (€/QALY)
**Scenario 1**					
General population	18.7	€35,497	€18,157	2.037	**€8914**
High-risk groups	16.7	€17,339			
**Scenario 2**					
General population	18.7	€35,497	€7733	1.038	**€7448**
Population with the highest anti-HCV prevalence plus high-risk groups	17.7	€27,764			

QALY, quality-adjusted life year; ICUR, incremental cost-utility ratio, defined as the difference in cost divided by the difference in health benefit when two strategies are compared

### Clinical benefits

**Fig 3:**
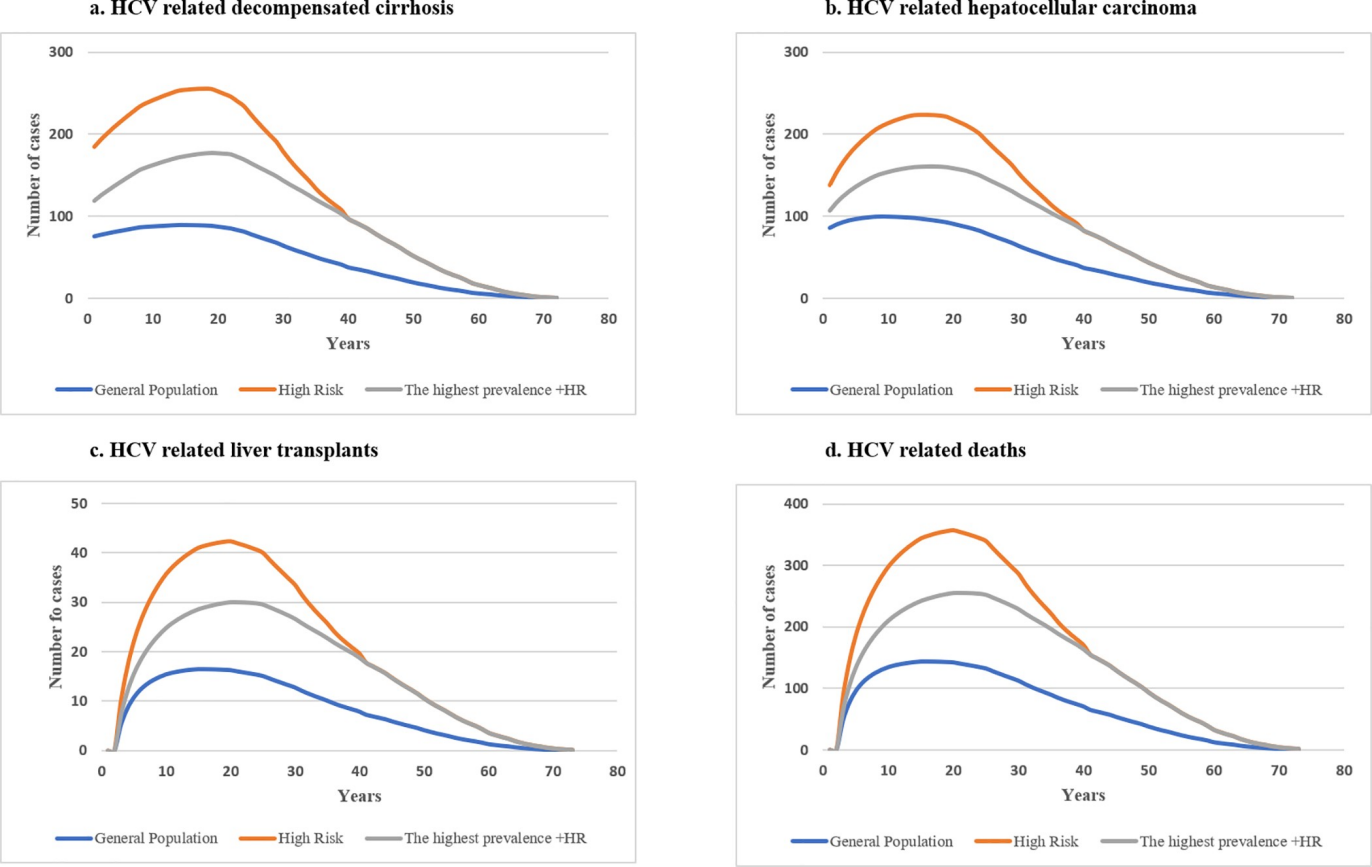
Annual impact on the number of clinical events with each population. HCV, hepatitis C virus; HR, High Risk. a. HCV related decompensated cirrhosis, b. HCV related hepatocellular carcinoma, c. HCV related liver transplants, d. HCV related death.

Over a lifetime horizon and without including discount rates, screening in the general population was associated with a significantly smaller number of patients developing DC and HCC, or LT requirement when compared to screening in the population with the highest anti-HCV prevalence plus high-risk groups, or screening high-risk groups (Fig 3A and 3D). In addition, screening in the general population led to a reduction in the total number of liver-related deaths relative to the other 2 approaches (Table 3).

**Table 3:** Number of liver-related complications avoided (discounted).

	DC	HCC	LT	HCV-related deaths
**Scenario 1**				
General population, N° cases	1845	2005	307	2693
High-risk groups, N° cases	4791	4116	701	5946
*N*° *cases avoided* ^*a*^	**-2946**	**-2111**	**-394**	**-3253**
**Scenario 2**				
General population, N° cases	1845	2005	307	2693
Population with the highest anti-HCV prevalence plus high-risk groups, N° cases	3665	3328	566	4846
*N*° *cases avoided* ^*a*^	**-1820**	**-1323**	**-259**	**-2153**

DC, decompensated cirrhosis; HCC, hepatocellular carcinoma, HCV, hepatitis C virus; LT, liver transplantation

^a^Negative values refer to cases avoided

### Sensitivity analysis

When comparing the general population to high-risk groups (scenario 1) or the group with highest anti-HCV prevalence plus high-risk groups (scenario 2), all results from the one-way sensitivity analyses confirmed the robustness of the base-case results, with all ICUR values under the accepted threshold [42, 43].

**Fig 4:**
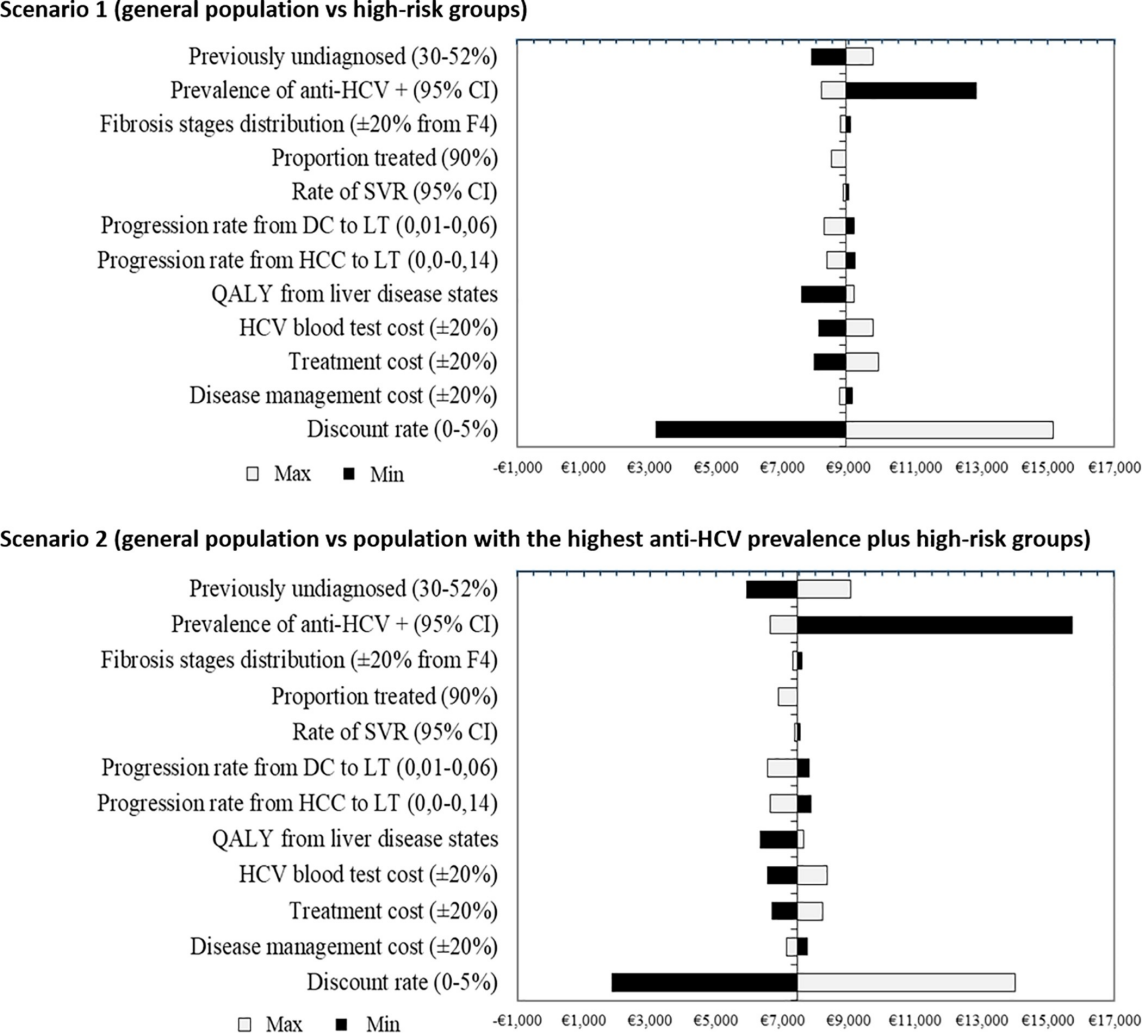
One-way sensitivity analyses. **Scenario 1 (general population vs high-risk groups) and Scenario 2 (general population vs population with the highest anti-HCV prevalence plus high-risk groups), using a tornado diagram.** ICUR for the upper and lower limits of each parameter examined are shown on the horizontal axis of the diagram.

The one-way sensitivity analyses are depicted in tornado diagrams. The ICUR values ranged from €7566 to €15,153 per QALY gained per patient in scenario 1 (Fig 4A) and from €5920 to €14,024 per QALY gained per patient in scenario 2 (Fig 4B). The most influential parameter in the analysis was the prevalence of anti-HCV antibodies. The ICUR increased as the prevalence of anti-HCV antibodies decreased. Similarly, decreases in the percentage of undiagnosed patients, utility values, and cost of hepatitis C antibody testing and treatment also led to increases in the ICUR. The analysis results were insensitive or only modestly sensitive to the other inputs included (Fig 4).

On the other hand, increasing the percentage of patients treated to 95% or 100%, would change the ICUR to €8249 or €8486 per QALY gained in scenario 1, and €6574 or €6298 per QALY gained in scenario 2, respectively. The ICUR decreased to €8423, €7933 and €7442 in scenario 1, and €7062, €6675 and €6289 in scenario 2, when drug costs were reduced by 10%, 20%, and 30%, respectively.

## Discussion

Hepatitis C infection generates a considerable social and economic burden, and constitutes a global public health problem [5]. The value and efficacy of the new DAAs has been amply proven in real-world chronic hepatitis C patients [44–46], but currently many individuals are still unaware that they have this infection. One objective of the WHO is to eliminate hepatitis C by the year 2030. To achieve this goal, treatment rates should increase to cover 90% of new diagnoses, and this can only be attained by screening strategies [47].

Many countries do not have universal screening programs, and testing is only done in persons belonging to risk groups for the infection. The results of this study show that this approach does not suffice to eliminate the infection, as is recommended by the WHO. More extensive screening strategies directed toward various populations are needed. The analysis carried out here was designed to determine what HCV screening strategy should be implemented in terms of efficiency to eliminate the virus. The results suggest that one-time HCV screening of the general population is cost-effective relative to screening of high-risk groups or populations with the highest anti-HCV prevalence plus high-risk groups. A strategy of chronic HCV screening and treatment for the general population would imply a substantial expenditure for the public health system. But this investment would result in detection and treatment of a larger number of infected individuals, which, in the long run, would provide considerable health benefits. Because of the demonstrated effectiveness of DAAs on real-world SVR rates [7], leading to cure in most cases, HCV-associated morbidity and mortality would decrease, the patient’s life expectancy would be extended and quality of life improved, and transmission of the virus to others would be greatly reduced [26].

Economics evaluations in other countries have shown that expanding HCV screening or screening and treatment in population groups or the general population is a cost-effective strategy [3, 48–54] Although these evaluations cannot be compared to the one reported here because of differences in the analytical approaches used, our findings are clearly consistent with those of the previous studies, as screening of the general population was the preferred policy option. Regarding the clinical outcome, the results with our model were also similar to those of studies on general population screening plus DAA therapy in terms of lowering the incidence of liver-related complications and improving the patients’ quality of life [50, 52–54].

This study has several limitations. First, there is a degree of uncertainty in the parameters related to the epidemiology of the infection (undiagnosed patients, HCV prevalence, HCV-RNA detection, and fibrosis stage at diagnosis). The latest data from studies in the Spanish population were included in the analysis, and we also examined the impact of modifying these parameters in one-way sensitivity analyses. These parameters were the most influential in the results, but in all the situations examined, the ICUR remained under the accepted threshold values in Spain.

Another parameter involving some uncertainty was the percentage of patients treated. In the base case, we assumed that 82% of patients diagnosed would be treated. However, considering the broad access to treatment implemented in Spain, the percentage of treated patients could be higher. To evaluate this factor, we performed a sensitivity analysis in which the percentage treated was increased to 90% and 95%, which yielded only a modest change in the ICUR, but a considerable clinical impact associated with the morbidity and mortality of the disease.

Finally, the cost of the antiviral regimens also implied uncertainty. Further sensitivity analyses using different DAA costs showed that the results were persistently robust and there was no significant impact on the ICUR.

Recent studies have shown that achieving HCV cure has benefits not only for the individual patient (better quality of life, reductions in extrahepatic manifestations of the infection), but also for society as a whole, such as increases in work productivity, reductions in HCV infectivity [55], and decreases in HCV management costs in the health system [56–58]. As our study did not take into account these factors, the potential benefits attained could be greater than those reported here. In the light of these advantages, some countries such as France have recommended HCV screening of the general population.

In conclusion, based on the results obtained in this analysis and from a public health perspective, HVC screening of the general population is an efficient approach that should be implemented in Spain with the aim of achieving elimination of this virus.

## Supporting information

S1 Text Calculation of the ICER per patient. (DOCX)

S2 Text Markov model specification. (DOCX)

S1 Fig Markov model scheme. The Markov model structure used to simulate the natural history of chronic hepatitis C.
(TIF)

S1 Table Markov model transition probabilities. (DOCX)

S2 Table Natural age-specific-group mortality Spanish population. (DOCX)
