# Supplementary material for: Healthcare value of implementing hepatitis C screening in the adult general population in Spain
Source: PLoS One. 2018 Nov 28;13(11):e0208036. doi: 10.1371/journal.pone.0208036 (PMC6261617; doi:10.1371/journal.pone.0208036)
Supplement: S1 Text — (DOCX) [file pone.0208036.s001.docx]

**S1. Text. Calculation of the ICER per patient**

Scenario 1. The ICER was calculated as the difference in total cost between the two screening strategies (General population -GP- vs. High-risk -HR- groups) divided by the difference in LYG or QALYs (by the benefit, measured in QALYs or LYG) between the two strategies.

$$ICUR= \frac{Total Cost \text{Screening GP}-Total Cost \text{Screening HR}}{QALYs \text{Screening GP}-QALYs \text{Screening HR}} = \frac{▲Cost}{▲QALYs}$$

$$ICER= \frac{Total Cost \text{Screening GP}-Total Cost \text{Screening HR}}{LYG \text{Screening GP}-LYG \text{ Screening HR}}= \frac{▲ Cost}{▲ LYG}$$

Scenario 2. The ICER was calculated as the difference in total cost between the two screening strategies (General population -GP- vs. The highest anti-HCV prevalence plus High-risk -HR- groups) divided by the difference in LYG or QALYs (by the benefit, measured in QALYs or LYG) between the two strategies.

$$ICUR= \frac{Total Cost \text{Screening GP}-Total Cost \text{Screening The highest anti-HCV prevalence + HR}}{QALYs \text{Screening GP}-QALYs \text{Screening The highest anti-HCV prevalence + HR}} = \frac{▲Cost}{▲QALYs}$$

$$ICER= \frac{Total Cost \text{Screening GP}-Total Cost \text{Screening The highest anti-HCV prevalence + HR}}{LYG \text{Screening GP}-LYG \text{ Screening The highest anti-HCV prevalence + HR}}= \frac{▲ Cost}{▲ LYG}$$
