# Supplementary material for: Healthcare value of implementing hepatitis C screening in the adult general population in Spain
Source: PLoS One. 2018 Nov 28;13(11):e0208036. doi: 10.1371/journal.pone.0208036 (PMC6261617; doi:10.1371/journal.pone.0208036)
Supplement: S2 Text — (DOCX) [file pone.0208036.s002.docx]

**S2 Text. Markov model specification**

The Markov model structure used to simulate the natural history of chronic hepatitis C. All patients entered in the model are distributed among fibrosis states. Only in the first cycle of the simulation, the patients who are treated could change to the SVR health states according to direct-acting antiviral (DAA) efficacy [1].

The following assumptions were applied in the model:

- The probability to change to the SVR health states applies only in the first cycle.
- To all health states was applied an overall mortality probability that was estimated from data from the Life Tables of the Spanish National Statistics Institute [2-3].
- The specific mortality probability on the Health States decompensated cirrhosis, Hepatocellular carcinoma, Liver Transplant and Post-Liver Transplant was added to overall mortality.
- Transition probabilities were applied to the patients who survive in each health state according to the next equation *P_i_a_= p_i_a_ (1- p_i___death_)*. Considering

*P_i_a_* :the patients who changes from state i to the state a,

*p_i_a:_* the transition probability to change from state i to state a and *p_i___death:_* the probability of death from state i.

1. Feld JJ, Jacobson IM, Hézode C, Asselah T, Ruane PJ, Gruener N, et al. Sofosbuvir and Velpatasvir for HCV Genotype 1, 2, 4, 5, and 6 Infection. N Engl J Med. 2015;373:2599-607.
2. Spanish Ministry of Health, Social Policy and Equality [Internet]. [Mortality by age-groups in Spain]; 2014 [cited 2017 Ago 10]. Available from: http://www.msssi.gob.es.
3. Spanish Ministry of Health, Social Policy and Equality [Internet]. [Hepatic mortality by age in Spain]; 2014 [cited 2017 Ago 10]. Available from: http://www.msssi.gob.es.
