# Supplementary figures and images for: Healthcare value of implementing hepatitis C screening in the adult general population in Spain
Source: PLoS One. 2018 Nov 28;13(11):e0208036. doi: 10.1371/journal.pone.0208036 (PMC6261617; doi:10.1371/journal.pone.0208036)

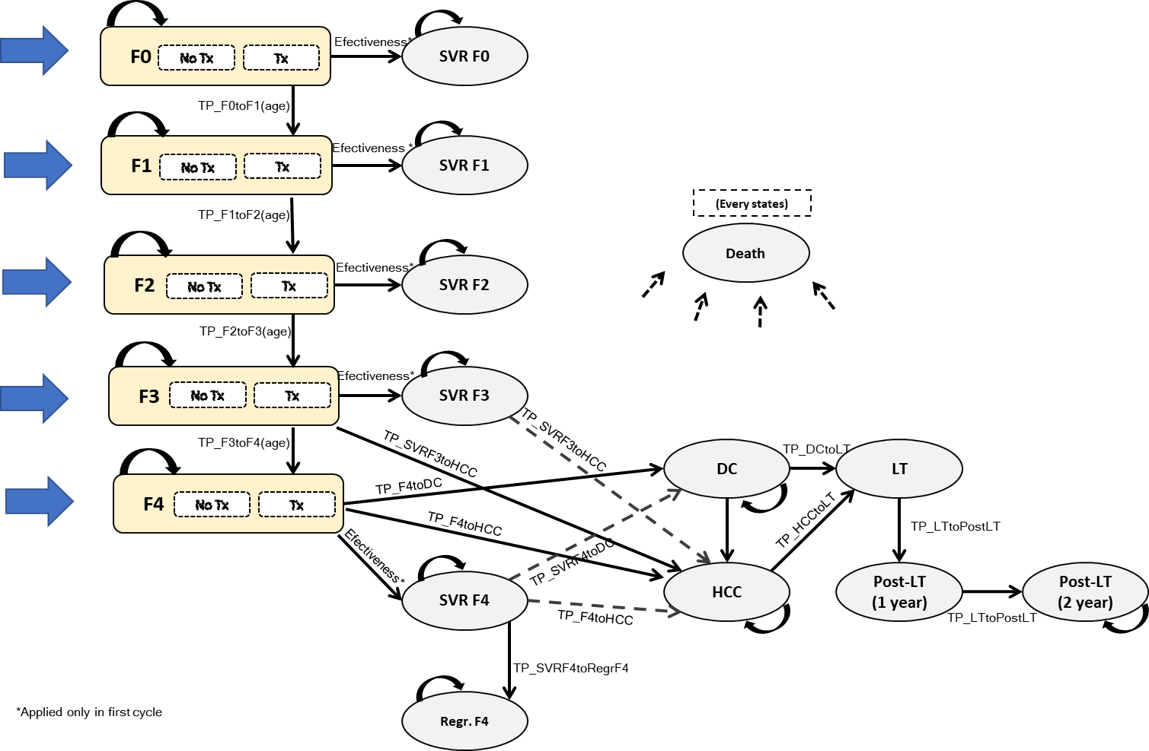

Supplement: S1 Fig — The Markov model structure used to simulate the natural history of chronic hepatitis C. (TIF) [file pone.0208036.s003.tif]
