# Supplementary material for: Healthcare value of implementing hepatitis C screening in the adult general population in Spain
Source: PLoS One. 2018 Nov 28;13(11):e0208036. doi: 10.1371/journal.pone.0208036 (PMC6261617; doi:10.1371/journal.pone.0208036)
Supplement: S1 Table — (DOCX) [file pone.0208036.s004.docx]

**S1 Table. Markov model transition probabilities**

| **Transition probability** | **Initial State** | **Final State** | **Base Case Value** |
| --- | --- | --- | --- |
|  |  |  |  |
| TP_F0toF1_20_29y | F0 | F1 (Age 20-29 years) | 0.314 |
| TP_F0toF1_30_49y |  | F1 (Age 30-49 years) | 0.131 |
| TP_F0toF1_50y |  | F1 (Age 50+ years) | 0.077 |
| TP_F1toF2_20_29y | F1 | F2 (Age 20-29 years) | 0.322 |
| TP_F1toF2_30_49y |  | F2 (Age 30-49 years) | 0.08 |
| TP_F1toF2_50y |  | F2 (Age 50+ years) | 0.074 |
| TP_F2toF3_20_29y | F2 | F3 (Age 20-29 years) | 0.22 |
| TP_F2toF3_30_49y |  | F3 (Age 30-49 years) | 0.133 |
| TP_F2toF3_50y |  | F3 (Age 50+ years) | 0.089 |
| TP_F3toF4_20_29y | F3 | F4 (Age 20-29 years) | 0.151 |
| TP_F3toF4_30_49y |  | F4 (Age 30-49 years) | 0.134 |
| TP_F3toF4_50y |  | F4 (Age 50+ years) | 0.088 |
| TP_F3toHCC |  | HCC | 0.011 |
| TP_SVRF3toHCC | F3 SVR | HCC | 0.00264 |
| TP_F4toDC | F4 | DC | 0.04 |
| TP_F4toHCC |  | HCC | 0.015 |
| TP_SVRF4toRegrF4 | F4 SVR | Regr. CC | 0.055 |
| TP_SVRF4toDC |  | DC | 0.00334 |
| TP_SVRF4toHCC |  | HCC | 0.00576 |
| TP_DCtoHCC | Decompensated cirrhosis | HCC | 0.068 |
| TP_DCtoLT |  | LT | 0.023 |
| TP_DCtoLiverDeath |  | Death | 0.138 |
| TP_HCCtoLT | Hepatocellular carcinoma | LT | 0.04 |
| TP_HCCtoLiverDeath |  | Death | 0.43 |
| TP_LTtoPostLT | Liver Transplant | Post-LT | 1.00 |
| TP_LTtoLiverDeath |  | Death | 0.21 |
| TP_PostLTtoLiverDeath | Post-Liver Transplant | Death | 0.057 |
