# Supplementary material for: Healthcare value of implementing hepatitis C screening in the adult general population in Spain
Source: PLoS One. 2018 Nov 28;13(11):e0208036. doi: 10.1371/journal.pone.0208036 (PMC6261617; doi:10.1371/journal.pone.0208036)
Supplement: S2 Table — (DOCX) [file pone.0208036.s005.docx]

**S2. Table. Natural age-specific-group mortality Spanish population**

| **Age group** | **Deaths*** | **Probability of death** |
| --- | --- | --- |
| **15-19** | 16.87 | 0,0001 |
| **20-24** | 28.125 | 0,0002 |
| **25-29** | 28.46 | 0,0002 |
| **30-34** | 40.071 | 0,0004 |
| **35-39** | 52.644 | 0,0005 |
| **40-44** | 99.182 | 0,0009 |
| **45-49** | 182.45 | 0,0018 |
| **50-54** | 299.05 | 0,0029 |
| **55-59** | 465.361 | 0,0046 |
| **60-64** | 692.904 | 0,0069 |
| **65-69** | 1018.772 | 0,0101 |
| **70-74** | 1594.2 | 0,0158 |
| **75-79** | 2821.453 | 0,0278 |
| **80-84** | 5115.987 | 0,0498 |
| **85-89** | 9693.939 | 0,0923 |
| **90-94** | 17311.872 | 0,1589 |
| **>95** | 26936.178 | 0,2361 |

*Deaths per 100,000 population. The probability of death was calculated according to cause of death disaggregated by age registered per 100,000 population.
